# Aortic Valve Interventions in Asymptomatic Severe Aortic Stenosis: Who, Why, and When?

**DOI:** 10.3390/jcm15031007

**Published:** 2026-01-27

**Authors:** Hilal Khan, Abdalazeem Ibrahem, Mohamed Farag

**Affiliations:** 1 Cardiothoracic Centre, Freeman Hospital, Newcastle-upon-Tyne NE7 7DN, UKmohamedfarag@nhs.net (M.F.); 2 School of Health, Medicine and Life Sciences, University of Hertfordshire, Hatfield AL10 9AB, UK

**Keywords:** aortic stenosis, asymptomatic, transcatheter aortic valve implantation, TAVI

## Abstract

Symptomatic severe aortic stenosis has an extremely high risk of death, ranging from 60 to 90% at five years if left untreated. This has informed the recommendation for urgent intervention upon diagnosis, especially when symptoms develop. Asymptomatic severe aortic stenosis has a four-year mortality between 30 and 50% if left untreated, which is similar to some metastatic cancers. Conservative management for patients with severe asymptomatic aortic stenosis was previously advocated, likely owing to the relative invasiveness of surgical aortic valve replacement. The advent of low-risk transcatheter aortic valve implantation with good medium-term durability has prioritized the need for a paradigm shift in the treatment of asymptomatic severe aortic stenosis towards a more proactive strategy of early intervention to reduce significant adverse events. This article provides a state-of-the-art overview of the contemporary management of patients with asymptomatic severe aortic stenosis.

## 1. Introduction

Aortic stenosis is the most common valvular heart pathology, accounting for 64% of deaths due to valvular heart disease globally [1]. It affects around 10% of octogenarians, and its prevalence increases with age [2,3]. There is significant morbidity and mortality associated with aortic stenosis, especially if left untreated. Registry data in patients with moderate and severe aortic stenosis report a four-year mortality of 45% and 44%, respectively [4]. Until recently, the management of severe aortic stenosis has been largely predicated on the presence of symptoms or a reduction in left ventricular systolic function.

Aortic stenosis is largely linked to three aetiologies: (1) senile calcific degeneration, (2) congenital bicuspid aortic valve, and (3) inflammatory rheumatic heart disease. The prevalence of rheumatic heart disease as a cause has decreased in developed countries due to the effective treatment of group A streptococcus infections [5]. Senile calcific degeneration is the leading cause in developed countries. It is mediated by a process similar to atherosclerosis. The valve leaflet is composed of a three-layer extracellular matrix covered by endothelial cells. The layer facing the ventricle is composed heavily of elastin fibres, which increases its flexibility. The spongiosa layer in the middle has a high proteoglycan content, which provides lubrication to the leaflet structure. Finally, the fibrosa layer facing the aorta is composed of collagen and fibroblasts, which support its role as the valve’s main load-bearing component. However, this is also the portion of the valve most susceptible to fibrocalcification. The main cell type in the leaflets is the valve interstitial cell (VIC). Endothelial injury on the valve surface stimulates the entry of low-density lipoproteins into the fibrosa, and these are oxidized by reactive oxygen species, recruiting monocytes and macrophages, which produce an inflammatory cascade that results in VIC releasing matrix metalloproteinases, causing fibrosis, eventually calcifying upon VIC apoptosis and the release of apoptotic bodies [6]. Congenital bicuspid aortic valve disease is triggered early in life by asymmetric leaflet motion and higher leaflet coaptation points, resulting in increased mechanical stress and driving the early onset of the inflammatory and calcific processes in these valve types [7,8].

The caveat with aortic stenosis management being driven by symptom-related presentations alone is that some patients will mask symptoms subconsciously by reducing activity levels to adapt to a haemodynamically significant aortic stenosis [9]. In certain cases, patients will only present with symptoms when the ejection fraction is below 50% and in florid heart failure. The onset of symptoms, such as chest pain, breathlessness, and syncope, heralds an alarming increase in mortality. In one of the original publications on aortic stenosis, the presence of angina was associated with a five-year survival of 39%, syncope of 30%, and heart failure of 0% [10].

The operative mortality risk from aortic valve surgery in the 1980s and earlier was around 7% [11]. In contemporary practice, the operative mortality risk of aortic valve replacement (AVR) for severe aortic stenosis is around 0.4% for younger patients and around 2.2% for octogenarians, reflecting substantial improvements in surgical techniques [12]. The advent of transcatheter aortic valve implantation (TAVI), particularly via the transfemoral route, has broadened access to aortic valve intervention even in patients with contraindications to surgery. In the original PARTNER trial, the 30-day mortality after TAVI was 5% in patients considered to have a risk of death from surgery that made the procedure inoperable [13]. This substantial reduction in procedure-related mortality, even in patients at the highest risk, heralded a new era of aortic valve intervention that could now be carried out via the percutaneous route with low procedural risk. In subsequent PARTNER trials, the 30-day death from any cause was 3.9% in the transcatheter aortic valve arm and 4.1% in the surgical intervention arm in intermediate surgical risk patients, and 0.4% in the transcatheter aortic valve arm and 1.1% in the surgical aortic valve replacement (SAVR) arm in low-risk patients [14,15]. Bicuspid aortic valves are less well represented in clinical trials of TAVI and present unique technical considerations, such as higher risk of paravalvular leak, device malposition, pacemaker implantation, and coronary access. This largely limits the expansion of TAVI indications to patients with bicuspid aortic valves until larger clinical trials are available [8].

This substantial reduction in procedural risk in the contemporary era, from a condition with a very high mortality and risk of adverse events in the longer term when untreated, provides an opportunity for a change in the current approach to the management of aortic stenosis based on symptomatology alone. There has already been a change in updated 2025 European guidelines reflecting this by providing a first-line recommendation for TAVI in symptomatic patients with tricuspid aortic valves who are 70 years or older, compared to 75 years in previous guidelines, as a result of increased procedural safety [16]. Notably, asymptomatic severe aortic stenosis has a deleterious effect on the myocardium, represented by subclinical myocardial fibrosis as well as sudden presentation with acute heart failure [17,18]. In view of these factors, the guidelines recommend considering early AVR in patients with asymptomatic severe aortic stenosis who are at low procedural risk [16].

## 2. Diagnostic Criteria and Evaluation of Haemodynamically Significant Aortic Stenosis

The current strong indication for AVR is based on the presence of symptoms or a reduction in ejection fraction attributed to haemodynamically significant aortic stenosis [16]. The assessment of severe aortic stenosis is largely based on echocardiographic criteria, primarily a valve area of less than or equal to 1 cm^2^, a peak aortic jet velocity of more than or equal to 65 mm Hg and a mean transvalvular aortic valve gradient of more than or equal to 40 mm Hg [9,16]. This type of aortic stenosis is referred to as high-gradient aortic stenosis, and it is given a class 1 recommendation for intervention when symptomatic [16]. There are, however, other entities that can present with reduced peak or mean transvalvular gradients but still have a significantly reduced aortic valve area. Firstly, a low-flow, low-gradient state due to severe left ventricular systolic dysfunction, and secondly, the more difficult-to-diagnose entity of paradoxical low-flow, low-gradient state with normal ejection fraction. Low flow has largely been defined as a stroke volume index of less than 35 mL/m^2^ [19]. Distinguishing patients with low-flow, low-gradient severe aortic stenosis from pseudo-severe aortic stenosis is largely performed by dobutamine stress echocardiography [20]. It is, however, difficult to differentiate these patient groups if there is no contractile reserve. In such cases, multimodal imaging with computed tomography (CT) of the aortic valve and an Agatston calcium score can help guide decision-making about whether a stenosis is severe or pseudo-severe. Calcium scores greater than 1200 Agatston units in women and 2000 Agatston units in men are considered supportive of significant aortic valve stenosis [21,22,23]. In addition, cardiac magnetic resonance imaging can provide ancillary information regarding the etiology of left ventricular systolic dysfunction in these patients. Furthermore, it can be helpful in patients with preserved ejection fraction in assessing the presence of fibrosis, which has prognostic implications, especially in asymptomatic severe aortic stenosis [24]. Patients with low-flow, low-gradient aortic stenosis with reduced ejection fraction, when symptomatic, are given a class 1 indication for intervention [16].

Paradoxical low-flow, low-gradient aortic stenosis with a normal or near-normal ejection fraction is a more difficult group of patients to identify. It is usually caused by the interplay of other factors, such as concentric left ventricular remodelling, impaired diastolic filling, atrial fibrillation, and/or mitral regurgitation, which can subtly affect left ventricular loading conditions and aortic valve gradients. CT of the heart and transthoracic echocardiography are usually key in making the diagnosis [25]. Nevertheless, in unclear cases, a careful evaluation of symptoms is crucial for determining whether the patient’s presentation is consistent with haemodynamically significant aortic stenosis. Symptomatic low-flow, low-gradient aortic stenosis with preserved ejection fraction is provided a class 2a indication for intervention in the current guidelines [16].

A more objective measure of assessing symptoms is by exercise testing. Exercise testing does require patients to be sufficiently mobile to be feasible, and this can be a major limiting factor, especially in the elderly [9]. Exercise testing with symptom-limited exercise protocols is largely safe in patients with asymptomatic severe aortic stenosis [26]. This has been the main method of testing for symptoms and the need for intervention in patients who are self-reportedly asymptomatic with severe aortic stenosis. Of note, there is significant heterogeneity between the studies reporting on exercise testing to evaluate for symptoms in severe aortic stenosis, but positive criteria are largely agreed to be the occurrence of limiting symptoms on low workload, a drop in blood pressure or less than 20 mm Hg rise in systolic blood pressure, sustained ventricular arrhythmias, or ST depression of more than 5mm on the electrocardiogram [27,28,29,30]. Some studies reported ST depression of more than 1 mm, and others of 3 mm as a positive test. Some reported ventricular ectopics as a sign of a positive test. Further, some reported a drop of 10 mm Hg or more in systolic blood pressure as a sign of a positive test, while others reported any drop in systolic blood pressure as being positive [9]. Additionally, the mean age of patients included in these studies ranged from 50 to 69 years, and these findings have not been validated in older patient groups.

International guidelines, namely, the European Society of Cardiology (ESC) and the American Heart Association (AHA), provide recommendations for intervention in asymptomatic, very severe aortic stenosis. The ESC guidelines recommend AVR in asymptomatic patients, when there is very severe high-gradient aortic stenosis (Vmax > 5 m/s or mean gradient ≥ 60 mmHg), rapid progression (severe calcification confirmed by Agatston score on CT and increase in Vmax ≥ 0.3 m/s/year), markedly elevated brain natriuretic peptide (BNP)/N-terminal pro-B-type natriuretic peptide (NT-proBNP) (three times more than the age- and sex-corrected normal range, confirmed on repeated measurements), left ventricular ejection fraction less than 55% without any other cause, and a drop in systolic blood pressure > 20 mmHg on exercise treadmill testing (ETT), and they also recommend considering AVR in patients with severe high-gradient aortic stenosis with a normal exercise test when the procedural risk is low [16]. The AHA guidelines for asymptomatic patients with normal ejection fraction recommend considering early intervention with SAVR only for very high-gradient severe aortic stenosis, rapid progression in gradient, BNP/NT-proBNP levels three times the normal range, abnormal exercise response during ETT, and patients who are low surgical risk [31]. The AHA guidelines do, however, recommend considering TAVI in symptomatic severe aortic stenosis in patients ≥65 years of age if the anatomy is suitable for TAVI [31].

## 3. Benefits of Intervention in Symptomatic Patients

Since the first SAVR were performed in patients with symptomatic severe aortic stenosis, there has been an improvement in long-term survival, although not always a complete restoration of life expectancy [32,33,34]. This is considered the driving factor behind the expansion of AVR procedures for the treatment of symptomatic severe aortic stenosis. The perioperative risk of SAVR is low, around 0.4% in younger patients, but rises with age [12] (Table 1). Surgical registry data have shown a significant operative mortality risk from AVR in patients 80 years or older. Ho et al. reported an 8.3% 30-day mortality risk after surgical aortic valve replacement in this age group [35]. Older data in octogenarians quoted a 17% 30-day mortality risk post-operatively and considered this to be an acceptable risk profile at the time for treating symptomatic severe aortic stenosis as opposed to the very high risk of mortality when untreated [36].

The advent of TAVI has altered this paradigm of procedural risk. In the original PARTNER trial, in patients with high or inoperable surgical risk, transcatheter procedures were associated with a 5% mortality risk, mainly in an octogenarian population [13]. Subsequent PARTNER trials 2 and 3 showed similarly low procedural risks of 3.9% and 0.4%, respectively, with TAVI (Table 1) [14,15]. A meta-analysis of six low-risk trials showed improved short-term survival with TAVI compared to SAVR, although longer-term data are needed to better understand the durability of transcatheter valve replacement in younger patients [46]. Reassuringly, mid-term TAVI data show a potentially lower risk of structural valve deterioration in patients with transcatheter aortic valves—15.4% compared to up to 20.8% in some surgical aortic valves at 10 years (Table 2) [47]. Overall, at least from the limited data available, transcatheter aortic valves appear to have similar 10-year durability to surgical aortic valves [48]. The seven-year follow-up of the low-risk PARTNER 3 trial did not show any differences in outcomes or valve failure rates between the surgical and TAVI groups [38]. The longer-term durability is not yet known.

## 4. The Evidence for Intervention in Asymptomatic Patients

There is growing interest in earlier intervention in severe aortic stenosis, driven by data on elevated five-year mortality in untreated moderate aortic stenosis, likely due to its progression to severe aortic stenosis over time [56]. The emergence of lower-risk TAVI procedures with good medium-term durability drives a shift in perspective in the management of aortic stenosis [53].

There have been four randomized controlled trials in asymptomatic severe aortic stenosis that suggested a benefit towards early intervention. The RECOVERY trial (Early Surgery or Conservative Care for Asymptomatic Aortic Stenosis) showed a lower risk of 30-day operative mortality or death from cardiovascular causes over the follow-up period of 4 years in the early surgical intervention group (1% of patients died compared to 15% in the conservative intervention group) (Table 3). This would suggest a significant risk of sudden death in untreated asymptomatic severe aortic stenosis or perhaps less favourable operative outcomes upon the emergence of symptoms [57]. There was also a reduction in stroke and heart failure hospitalizations with early surgery compared to conservative care (1% vs. 4% and 0% vs. 11%, respectively). It is possible that some patients were not truly asymptomatic, as patients were selected based on the absence of reported symptoms, and only those with non-specific symptoms underwent ETT.

More interestingly, the results of the AVATAR trial (Aortic Valve Replacement Versus Conservative Treatment in Asymptomatic Severe Aortic Stenosis) showed a reduction in the primary endpoint, a composite of all-cause mortality, acute myocardial infarction, stroke, and unplanned heart failure hospitalization requiring intravenous diuretic or inotrope treatment. This occurred in 15% of the early intervention group and 35% of the conservative treatment group over three years (Table 3). Of note, the trial was stopped early because the prespecified number of events had been reached in the conservative treatment arm. All patients in this study were screened for asymptomatic status by ETT. These results reflect that, even in patients who have negative exercise stress tests, there is a risk of increased adverse events if asymptomatic severe aortic stenosis is left untreated [58,61].

In the EVOVLED trial (Early Intervention in Patients With Asymptomatic Severe Aortic Stenosis and Myocardial Fibrosis), there was no difference in the primary endpoint, which was a composite of all-cause mortality or unplanned aortic stenosis-related hospitalization during the mean follow-up period of 42 months; however, there was a higher rate of unplanned hospitalization for heart failure in the conservative arm [59]. Furthermore, there were fewer strokes in the early intervention arm compared to conservative management (7% vs. 13%), which may suggest a protective effect of early intervention on stroke risk compared to the aortic stenosis itself. This study included only patients with adverse remodelling, as defined by late gadolinium enhancement on cardiac magnetic resonance imaging and elevated biomarkers. These are all adverse prognostic features, and it may be that this is why, unlike in the previous two studies, which demonstrated a reduction in mortality, this study did not show a reduction. Another factor is perhaps the lack of mandated negative exercise testing to ensure all patients were truly asymptomatic.

The largest study in this patient group is the EARLY TAVR trial (Transcatheter Aortic-Valve Replacement for Asymptomatic Severe Aortic Stenosis). Patients were deemed truly asymptomatic based on negative exercise testing before inclusion. This study showed a reduction in the composite primary endpoint of death from any cause, stroke, or unplanned hospitalization for cardiovascular causes of 27% in the early intervention arm compared to 45% in the conservative treatment arm. This was mainly driven by a reduction in unplanned admissions for heart failure and a reduction in stroke in the early transcatheter intervention arm. However, there was no reduction in deaths from any cause [60].

A recent pooled analysis of these studies suggests that most of the signal of benefit from early intervention may arise from the effectiveness of clinical surveillance, coupled with crossover to AVR. The AVATAR trial has the lowest crossover to AVR (44%) and the highest mortality in the conservative treatment group. Whereas the EARLY TAVR trial had the highest crossover to AVR at 87%. The annual crossover rates in the EVOLVED and EARLY TAVR trials were 22% and 22.9%, respectively [62]. The only caveat is that such close monitoring in a clinical trial may allow the feasibility of rapid intervention when needed in a conservatively treated patient group. Many public healthcare systems around the world, including in Europe, cannot offer rapid intervention due to rising healthcare pressures to react efficiently to changes in a patient’s symptomatology in a timely manner [63].

The EASY-AS trial (The Early valve replacement in severe ASYmptomatic Aortic Stenosis) is an ongoing study that will provide further insight into the management of this patient population [64].

## 5. Discussion and Future Directions

**Figure 1:**
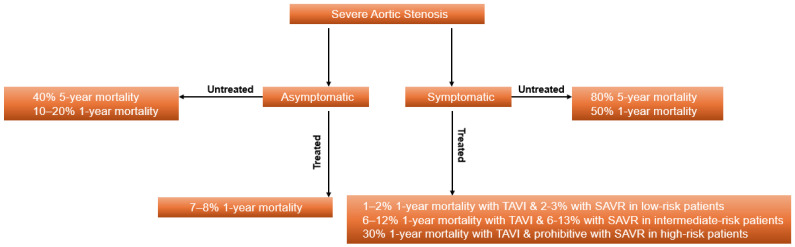
Prognosis of patients with severe aortic stenosis depending on symptoms and intervention status.

There is currently no doubt regarding the need to treat symptomatic severe aortic stenosis due to its significant mortality. The mortality rate, when untreated, is 20% over two years and around 44% at four years [4,65,66]. In octogenarians, the mortality risk can be as high as 90% at five years, when untreated [67]. This leaves little debate for delaying treatment in symptomatic severe aortic stenosis (Figure 1). There has been growing interest in intervening early to prevent mortality and acute valve syndrome in asymptomatic severe aortic stenosis in a similar manner to mitral valve regurgitation, driven by the evidence that this restores life expectancy and prevents unplanned admissions with heart failure hospitalizations. The updated European guidelines therefore recommend considering early intervention in asymptomatic severe aortic stenosis at low procedural risk [16].

### 5.1. Role of Advanced Imaging in Screening High-Risk Asymptomatic Patients with Severe Aortic Stenosis

It may be that the discrepant results in studies of early intervention in asymptomatic severe aortic stenosis are due to failure to identify high-risk asymptomatic patients, or to potentially including symptomatic patients if no screening test is performed. ETT is not always feasible in an older age population [9]. Stress echocardiogram may have a role as the new standard of care for identifying patients who become symptomatic under pharmacological stress [68]. In addition, the use of global longitudinal strain may help identify higher-risk patients with severe aortic stenosis [69]. Furthermore, cardiac magnetic resonance imaging may play a role in identifying fibrosis, a recognized poor prognostic feature in severe aortic stenosis [18]. Biomarkers, such as BNP, play a role in identifying higher-risk patients who are displaying physiological markers of stress due to severe aortic stenosis. Studies have shown elevated BNP as predictive of increased mortality in aortic stenosis [70]. These features, which are associated with poor longer-term outcomes, should guide the prioritization of early AVR for patients with asymptomatic severe aortic stenosis.

### 5.2. Risk of Sudden Death and Acute Valve Syndrome in Asymptomatic Severe Aortic Stenosis

Real-world observational data in over 70,000 patients with aortic stenosis show a significant mortality rate of 33% at four years in untreated moderate aortic stenosis, 45% in moderate-severe aortic stenosis, and 44% in severe aortic stenosis [4]. In this cohort, a multivariate analysis of all-cause mortality showed that both moderate-severe and severe aortic stenosis have a significantly higher risk of mortality than all other variables, such as age, gender, chronic obstructive pulmonary disease, stroke, and coronary artery disease, with the only exception being metastatic cancer [4].

The original recommendation for watchful waiting in moderate-severe aortic stenosis comes from small single-centre studies in which almost half of the patients became symptomatic within 1–2 years and needed urgent surgery [71]. In the asymptomatic cohort that did not have surgery, over 50% died at five years [66,71]. These initial studies were compared to a reference population from the United States that reported an excess risk of sudden cardiac death of less than 1% per year in untreated patients. Of note, the populations included in the conservative arm of these studies were two decades younger.

### 5.3. Procedural Risk of Aortic Valve Replacement for Haemodynamically Significant Aortic Stenosis

The significant five-year mortality of between 30 and 50% in untreated asymptomatic moderate or severe aortic stenosis makes the procedural risks appear negligible in comparison [4,56]. In the RECOVERY trial, there was no increased 30-day mortality with early SAVR [57]. Similarly, in the PARTNER 3 trial, the 30-day risk of stroke or death was 1% with TAVI and 3.3% with surgical aortic valve replacement [15]. In the original PARTNER trial, in a predominantly octogenarian cohort, procedural mortality was 5%, given that this was performed by operators with limited experience with a novel transcatheter technique at the time. The overall risk calculus in the contemporary era lends itself favourably towards early intervention when otherwise asymptomatic patients with severe aortic stenosis can have a mortality of between 30 and 50% at four years [4,13,56]. Of note, the five-year mortality for metastatic breast cancer is around 60% in observational data, whereas the four-year mortality for asymptomatic moderate-severe aortic stenosis is almost 50% [4,56,72]. This leads one to question why asymptomatic stenosis is not treated in a similar manner to early stage cancer, with prompt treatment to prevent adverse events.

### 5.4. Long-Term Durability of Bioprosthetic Aortic Valve Replacement

One of the reasons to delay surgery until patients are symptomatic is concerns of bioprosthetic valve failure and the need for repeat interventions. The risk of early valve failure is low for both surgical and transcatheter aortic valves. Surgical bioprosthetic valves have a 12% redo operative rate over 15 years in one registry [73]. Medium-term data for TAVI of almost 10 years showed that only 6.5% of transcatheter valves developed significant deterioration. Recent seven-year follow-up data showed rates of 6.9% in the TAVI arm and 7.3% in the surgical arm (Table 3) [53]. These durability data are very reassuring, at least for TAVI among patients in their seventh and eighth decades of life.

## 6. Conclusions

Aortic stenosis is associated with a significantly elevated risk of death, which is further increased by the presence of symptoms. Patients with untreated asymptomatic moderate to severe aortic stenosis have mortality rates not much lower than those of symptomatic severe aortic stenosis in contemporary large registry data. Modern transcatheter or surgical aortic valve replacements in appropriate patient groups carry a low procedural risk compared with conservative management, with excellent medium-term durability. Mortality rates of asymptomatic, untreated significant aortic stenosis are comparable to some cancers, and a paradigm shift towards earlier detection and intervention, especially with the availability of lower-risk transcatheter interventions, should be considered.

## Figures and Tables

**Table 1:** Randomized trials of transcatheter aortic valve intervention. TAVI—transcatheter aortic valve intervention; NR—not reported; STS—Society of Thoracic Surgeons Risk Score.

Study	Study Population	Sample Size (n)	TAVI Valve	30-Day Mortality	1-Year Mortality	Long-Term Mortality
PARTNER 1 [13,37]	High risk, inoperable	TAVI 179 Conservative management 179	Edwards SAPIEN	5.0% 2.8%	30.7% 49.7%	71.8% at 5 years 93.6% at 5 years
PARTNER 2A [14]	Intermediate risk, STS score 4–8%	TAVI 1011 Surgery 1021	Edwards SAPIEN XT	3.9% 4.1%	12.3% 12.9%	46.0% at 5 years 42.1% at 5 years
PARTNER 3 [15,38]	Low risk, STS score < 4%	TAVI 496 Surgery 454	Edwards SAPIEN 3	0.4% 1.1%	1.0% 2.5%	19.5% at 7 years 16.8% at 7 years
SURTAVI [39]	Intermediate Risk, STS score 3–15%	TAVI 864 Surgery 796	CoreValve/Evolut	2.2% 1.7%	6.7% 6.8%	30.0% at 5 years 28.7% at 5 years
EVOLUT LR [40]	Low risk, STS score < 3%	TAVI 725 Surgery 678	CoreValve/Evolut	0.5% 1.3%	2.4% 3.0%	13.5% at 5 years 14.9% at 5 years
NOTION [41]	Low risk, STS score 3%	TAVI 145 Surgery 135	CoreValve	2.9% 5.2%	5.0% 7.4%	62.7% at 10 years 64.0% at 10 years
NOTION 2 [42,43]	Low risk, STS score < 4%	TAVI 187 Surgery 183	Evolut/SAPIEN/Portico/ Accurate Neo/Lotus/Myval	0.0% 0.8%	1.5% 0.8%	3.8% at 3 years 2.8% at 3 years
UK TAVI [44]	Intermediate risk or high risk	TAVI 458 Surgery 455	Any CE marked TAVI valve	1.8% 0.9%	4.6% 6.6%	NR
DEDICATE-DZHK6 [45]	Low and intermediate risk	TAVI 701 Surgery 713	Any CE marked TAVI valve	0.6% 1.8%	2.6% 6.2%	NR

**Table 2:** Comparison of durability data in transcatheter and surgical aortic valve bioprosthesis. TAVI: Transcatheter Aortic Valve insertion.

Study	Sample Size (n)	Follow-Up (Years)	Valve Type	Structural Valve Deterioration Requiring Intervention (%)	Structural Valve Deterioration (%)
Jamieson et al., 2005 [49]	1823	18 years	Carpentier-Edwards, surgical aortic valve	7.2%	25% at 15 years 35% at 18 years
Yankah et al., 2008 [50]	1513	12 years	Mitroflow surgical valve	4.2%	14% at 10 years 31% at 12 years
Forcillo et al., 2013 [51]	2405	20 years	Carpentier-Edwards, surgical aortic valve	3.7%	4% at 10 years 33% at 20 years
Guenzinger et al., 2015 [52]	455	20 years	St Jude Medical Biocor	8.1%	8% at 10 years 33% at 20 years
Sathananthan et al., 2021 [53]	235	10 years	Cribier-Edwards (20.9%) Edwards SAPIEN (77.4%) CoreValve (1.7%)	2.6%	6.5% at 10 years
NOTION [41]	TAVI 145 Surgery 135	10 years	CoreValve Any surgical valve	1.5% 10%	15.4% at 10 years 20.8% at 10 years
Elbasha et al., 2024 [54]	302	10 years	CoreValve	1.0%	2.1% at 10 years
Dietze et al., 2025 [47]	1825	10 years	Corevalve Edwards SAPIEN	0.1% 0.1%	2.8% at 10 years 20% at 10 years
SURTAVI [39]	TAVI 879 Surgery 867	5 years	Corevalve/Evolut R Any surgical valve	3.5% 1.9%	
PARTNER 2 [55]	TAVI 1011 Surgery 1021	5 years	Edwards SAPIEN XT Any surgical valve	4.7% 1.3%	9.5% at 5 years 3.5% at 5 years
PARTNER 3 [38]	TAVI 496 Surgery 454	7 years	Edwards SAPIEN 3 Any surgical valve	6.7% 6.0%	7.3% at 7 years 7.6% at 7 years
EVOLUT LR [40]	TAVI 725 Surgery 678	5 years	CoreValve/Evolut Any surgical valve	3.3% 2.5%	

**Table 3:** Randomized trial data on safety outcomes of aortic valve replacement in asymptomatic severe aortic stenosis. MACE: Major Adverse Cardiovascular Events; ETT: Exercise Treadmill testing; TAVI: Transcatheter Aortic Valve insertion.

Study	Study Population	Sample Size (n)	Valve Type	30-Day Mortality (%)	1-Year Mortality (%)	Stroke (%)	Heart Failure Hospitalization (%)	MACE (%)
RECOVERY [57]	Low risk, ETT negative	Early Surgery 73 Conservative 72	Surgical valve	0.0% 4.0%	7.0% 21.0%	1% 4%	0% 11%	NR
AVATAR [58]	Low risk, ETT negative	Early Surgery 78 Conservative 79	Surgical valve	1.4% 4.0%	7.7% 16.5%	4% 4%	4.01% 12.94%	15.22% 34.70%
EVOLVED [59]	Low risk, fibrosis on CMR	Early Intervention 113 Conservative 111	Surgical/TAVI	1.0% 0.0%	8.8% 5.4%	7% 13%	6% 17%	NR
EARLY-TAVR [60]	Low risk, ETT negative	TAVI 455 Conservative 446	Edwards SAPIEN	0.2% 0.0%	8.4% 9.2%	4.2% 6.7%	20.9% 41.7%	NR

## Data Availability

No new data were created or analyzed in this study. Data sharing is not applicable to this article.
